# Status and correlates of food and nutrition literacy among parents-adolescents’ dyads: findings from 10 Arab countries

**DOI:** 10.3389/fnut.2023.1151498

**Published:** 2023-05-02

**Authors:** Maha Hoteit, Rania Mansour, Hala Mohsen, Khlood Bookari, Fadwa Hammouh, Sabika Allehdan, Dalal AlKazemi, Haleama Al Sabbah, Hasnae Benkirane, Iman Kamel, Radwan Qasrawi, Reema Tayyem, the regional food literacy group

**Affiliations:** ^1^Food Science Unit, National Council for Scientific Research (CNRS), Beirut, Lebanon; ^2^PHENOL Research Group (Public Health Nutrition Program Lebanon), Faculty of Public Health, Lebanese University, Beirut, Lebanon; ^3^School of Social Sciences and Humanities, Doha Institute for Graduate Studies, Program of Social Work, Doha, Qatar; ^4^Department of Clinical Nutrition, Faculty of Applied Medical Sciences, Taibah University, Madinah, Saudi Arabia; ^5^National Nutrition Committee, Saudi Food and Drug Authority, Riyadh, Saudi Arabia; ^6^Department of Nutrition and Dietetics, Faculty of Health Sciences, American University of Madaba, Amman, Jordan; ^7^Department of Biology, College of Science, University of Bahrain, Zallaq, Bahrain; ^8^Department of Food Science and Nutrition, College of Life Sciences, Kuwait University, Kuwait City, Kuwait; ^9^Department of Public Health, College of Health Sciences, Abu Dhabi University, Abu Dhabi, United Arab Emirates; ^10^Joint Research Unit in Nutrition and Food, RDC-Nutrition AFRA/IAEA, Ibn Tofail University-CNESTEN, Kenitra, Rabat-Salé-Kénitra, Morocco; ^11^National Research Centre, Cairo, Egypt; ^12^Department of Computer Science, Al-Quds University, Jerusalem, Palestine; ^13^Department of Computer Engineering, Istinye University, Istanbul, Turkey; ^14^Department of Human Nutrition, College of Health Sciences, Qatar University, Doha, Qatar

**Keywords:** Arab countries, nutrition literacy, food literacy, adolescents, parents

## Abstract

**Background:**

Food literacy is capturing the attention worldwide and gaining traction in the Arab countries. Strengthening food and nutrition literacy among Arab teenagers are important promising empowering tools which can protect them from malnutrition. This study aims to assess the nutrition literacy status of adolescents with the food literacy of their parents in 10 Arab countries.

**Methods:**

This cross-sectional study involving a convenient sample of 5,401 adolescent-parent dyads (adolescents: mean age ± SD: 15.9 ± 3.0, females: 46.8%; parents: mean age ± SD: 45.0 ± 9.1, mothers: 67.8%) was launched between 29 April and 6 June 2022 in 10 Arab nations. The Adolescent Nutrition Literacy Scale (ANLS) and the Short Food Literacy Questionnaire (SFLQ) were used to meet the study aims.

**Results:**

More than one-quarter (28%) of adolescents had poor nutrition literacy, with 60% of their parents being food illiterate. The top three countries with nutritionally” less literate” adolescents were Qatar (44%), Lebanon (37.4%), and Saudi Arabia (34.9%). Adolescents’ age, gender, education level, primary caregivers, employment status, and the inclusion of nutrition education in the schools’ curriculum predicted the nutrition literacy levels of Arab adolescents. Besides, parental weight status, health status, parent’s food literacy level, and the number of children per household were significant determinants too. Adolescents studying at a university and having parents with adequate food literacy had the highest odds of being nutritionally literate (OR = 4.5, CI = 1.8–11.5, *p* = 0.001, OR = 1.8, CI = 1.6–2.1, *p* < 0.001, respectively).

**Conclusion:**

Nutrition literacy inadequacy among Arab adolescents is a prioritized challenge to be tackled.

## 1. Introduction

Malnutrition still rears its ugly head in many Arab countries, proposing that it is tremendously difficult for the region to meet the 2030 Agenda for Sustainable Development to reach “zero hunger” and eradicate all forms of malnutrition (1). High levels of food insecurity, malnutrition, and obesity is found in the Arab region, with 116 million people being food insecure, 43 million undernourished, and 115 million obese (2). Nonetheless, major inconsistencies among sub-regions do exist. Obesity is more prevalent in Gulf Cooperation Council (GCC); however, undernourishment is more of an issue in the least developed countries (LDCs) and Countries in Conflict (CICs) (3). Recent investigations show that the number of malnourished people in the Arab world increased from 4.8 million to 69 million between 2019 and 2020, accounting for nearly 16% of the population (4). In addition, the Eastern Mediterranean Region (EMR) is charaterized by its diversity into two main groups based on their Gross National Income (GNI): the oil producing high income countries and the low and medium income countries. Both countries are among the world’s most vulneable to the dire impacts of climate change. The EMR suffers from extreme fluctuations in tempratures and precipitation as well as natural water and agricultural land scracity (5). It is predicted that these patterns will worsen in the coming years, with climate change, with intense pressure on agriculture thus compounding the effect of climate change with addditional significant challenges to livelihoods and food security (5). A functional food systems literacy is therefore required to aid people in communicating and collaborating on food system problems, including food insecurity, within dynamic learning approach addressed mainly to resolve the threats induced by the “nutrition transition” phenomena in the EMR. For instance, due to the “nutrition transition,” adolescent obesity has reached a critical level in the Arab countries (6). Ensuring resilient food systems in the Arab world is challenging amidst the overwhelmed population growth, dwindling natural resources, and import dependency (7). Further, the COVID-19 crisis revealed how unprepared the region was to respond appropriately to the pandemic with food supply chains varying in complexity and vulnerable to disruption amongst sub-regions (8). Given these constraints, a multisector approach considering each policy’s situation in the larger food environment and supply chain is required. Arab countries must invest in sectors having long-lasting effects, particularly education. In other words, equipping Arab people with sufficient nutrition knowledge could help meet a range of sustainable nutrition goals. Nutrition education works best in unique intervention points in the life cycle, particularly throughout adolescence (9). Adolescence, a period of life ranging from 10-to 19 years old (10), is an ideal time to plan, apply, and monitor nutrition interventions. Inadequate nutrition in adolescence can potentially retard growth and sexual maturation and displaces adolescents at high risk of developing chronic diseases (11). Referring to the Nutbeams’ model in “health literacy,” (12) two emerging concepts, “food literacy” and the “nutrition literacy,” have grabbed the researchers’ attention since 1990, who provided plenty of definitions for these two terms. Food literacy is beyond nutrition knowledge; it includes skills, and behaviors, from knowing where food comes from to the ability to select and prepare these foods appropriately (13). On the other hand, nutrition literacy is the ability of individuals to obtain, process, and understand the basic nutrition information they need to make appropriate nutrition decisions (14). The latter has been linked to improved diet quality, nutrient adequacy, food label reading, and food security (15–17). Upon this, Arab school is an ideal destination for creating synergies to contribute to sustainable development by offering a unique chance for the formal education system to improve students’ nutrition literacy (18). All school-based activities advocating healthy eating, not only those held in the classroom, are part of an extended nutrition education curriculum known as a “macro-curriculum” (19). To date, the number of studies on adolescent nutrition literacy in the Arab world is limited. Therefore, we conducted this study to be the first to evaluate the nutrition literacy status of adolescents with the food literacy of their parents in ten Arab countries. The hypothesis anticipated in this study is that Arab adolescents express low levels of nutrition literacy, stressing the need to reach a regional consensus to integrate nutrition education into Arab schools’ curricula.

## 2. Methods

### 2.1. Study design and eligibility criteria

A cross-sectional study using the snowball sampling method was conducted in Lebanon and nine other Arab nations: Bahrain, Egypt, Jordan, Kuwait, Morocco, Palestine, Qatar, Saudi Arabia, and United Arab Emirates (UAE). A self-administered questionnaire (82 items)^1^ was disseminated to be completed by eligible adolescent-parents’ dyads and kept open between 29 April and 6 June 2022. Calls for participation were performed via social network platforms and the research teams’ networks. In each country, a convenient sample of parent’s-adolescents dyads were collected. Research assistants were sending the links for eligible participants, based on the following eligibility criteria: (i) the adolescent is between 10 and 19 years old; (ii) either parent/caregiver (age more than 18 to 85 years old); (iii) having nationality of the mentioned ten countries; and consenting to participate. Besides, we aimed to reach only one adolescent per household. Overall, 5,401 adolescent-parents’ dyads completed the survey and their data were considered for analysis.

### 2.2. Explanatory variables

The explanatory variables were as the follows: (i) demographic and socio-economic status of adolescents (age, gender, country of nationality, education level, working status, primary caregiver, parental education level, self-reported body weight and height), and whether nutrition education is a part of school’s curriculum; (ii) demographic and socio-economic status of parents (age, gender, country of nationality, marital status, education level, spouse’s education level, job type, number of co-residents per household excluding newborns, number of rooms excluding the kitchen and bathrooms, number of children, self-reported body weight and height, and health status). The body mass index (BMI) was calculated and evaluated per WHO recommendations (20).

### 2.3. Outcome variables

#### 2.3.1. Nutrition literacy

The Adolescent Nutrition Literacy Scale (ANLS), developed by Bari (21), was used to assess adolescents’ nutrition literacy. It compromised of 22 items under three sub-sections: Functional Nutrition Literacy (FNL) (7 items); Interactive Nutrition Literacy (INL) (6 items); Critical Nutrition Literacy (CNL) (9 items). Each item has a score range of 1–5. The scoring criteria is as the follows:

Total Nutrition Literacy (TNL): the sum of FNL, INL, and CNL (minimum-maximum score: 22–110; ≥ 66 is an average score).

FNL (questions 1–7): minimum-maximum score: 7–35 (≥ 21 is an average score).

INL (questions 8–13): minimum-maximum score: 6–30 (≥ 18 is an average score).

CNL (questions 14–22): minimum-maximum score: 9–45 (≥ 27 is an average score).

#### 2.3.2. Food literacy

Parental food literacy was evaluated using the Short Food Literacy Questionnaire (SFLQ), developed by Gréa Krause et al. (22). It consists of 12 items (score range: 7 to 52; average score ≥ 36), representing the functional (6 items), interactive (2 items), and critical (4 items) food literacy dimensions.

### 2.4. Ethical considerations

The study was performed based on the ethical standards laid down in the Helsinki Declaration. We obtained written approval from the Ethics Committee of Al Zahraa University Medical Center (ZhU#17, 2022), Beirut, Lebanon, and the universities from all participating countries. A consent form was added to the survey, informing participants about their, rights and confidentiality. The participation was entirely voluntary with no obligation to do so.

### 2.5. Statistical analysis

We performed the statistical analysis using the Statistical Package of Social Sciences Software (SPSS) (Version 25.0. IBM Corp: Armonk, NY, USA). A “weighting” variable was created to adjust the representation of the sampled population. Respondents’ characteristics were presented as frequencies (percentages) for categorical variables, while means ± standard deviation (SD) for continuous variables. The adolescence stages were classified as follows: early adolescence (10–13 years old), middle adolescence (14–16 years old), and late adolescence (17–19 years old). The normality of data was checked using the Shapiro–Wilk test. Due to the non-normal distribution, Kruskal-Wallis test was used to determine score differences according to country. Chi-squared test (*χ*2) was used to determine associations between study variables. In addition, the binary backward stepwise regression was used to examine the predictors of adolescents’ nutrition literacy. A value of *p* of 5% was considered significant.

## 3. Results

### 3.1. General characteristics of adolescents and their parents, overall and by gender

A total of 5,401 adolescent-parent dyads participated in this study. Table 1 shows the general characteristics of participants. Regarding the nationality, 11% were from Lebanon, 9.4% from Bahrain, 12.2% from Egypt, 10.4% from Jordan, 8.9% from Kuwait, 10.9% from Morocco, 9.6% from Palestine, 10.2% from Qatar, 9.1% from Saudi Arabia, and 8.3% from UAE.

**Table 1:** General characteristics of adolescents, overall and by gender.

Variables	Overall (*N* = 5,401)		Male (*n* = 2,872; 53.2%)		Female (*n* = 2,529; 46.8%)		Value of *p*
Adolescent Participant	Mean ± SD		Mean ± SD		Mean ± SD		
Age (Year)	15.9 ± 3.0		15.0 ± 3.0		16.0 ± 3.0		<0.001
	*N*	%	*n*	%	*n*	%	
Adolescence stage							<0.001
Early adolescence (10–13 years old)	1,354	25.1	898	31.3	456	18.0	
Middle adolescence (14–16 years old)	1,299	24.0	742	25.8	557	22.0	
Late adolescence (17–19 years old)	2,748	50.9	1,232	42.9	1,516	59.9	
Country of nationality							<0.001
Lebanon	594	11.0	374	13.0	220	8.7	
Bahrain	507	9.4	232	8.1	274	10.9	
Egypt	657	12.2	477	16.6	180	7.1	
Jordan	563	10.4	325	11.3	239	9.4	
Kuwait	483	8.9	195	6.8	288	11.4	
Morocco	590	10.9	377	13.1	212	8.4	
Palestine	517	9.6	251	8.7	267	10.5	
Qatar	549	10.2	298	10.4	251	9.9	
Saudi Arabia	491	9.1	206	7.2	285	11.3	
United Arab Emirates	450	8.3	137	4.8	313	12.4	
Weight status (*n* = 5,364)							<0.001
Underweight	263	4.9	98	3.4	165	6.4	
Normal-weight	3,471	64.7	1863	65.3	1,608	64.0	
Overweight	1,451	27.0	771	27.0	680	27.0	
Obese	179	3.4	120	4.2	59	2.2	
Education level							<0.001
Not attending school	19	0.3	5	0.2	14	0.6	
Elementary school level	752	13.9	506	17.6	246	9.7	
Intermediate school level	1,133	21.0	692	24.1	441	17.5	
Secondary school level	1816	33.6	1,030	35.9	786	31.1	
University level	1,681	31.1	639	22.2	1,042	41.2	
Primary caregiver							<0.001
Both parents	4,650	86.1	2,472	86.1	2,178	86.1	
Father only	143	2.6	86	3.0	57	2.3	
Mother only	447	8.3	260	9.1	187	7.4	
Others	147	2.7	49	1.7	98	3.9	
None (living alone)	14	0.3	5	0.2	9	0.4	
Currently working							<0.001
No	4,782	88.5	2,478	86.3	2,304	91.1	
Yes	619	11.5	394	13.7	225	8.9	
Education level of mother							<0.001
Never attend school	331	6.1	194	6.8	136	5.4	
Elementary school level	398	7.4	201	7.0	197	7.8	
Intermediate school level	453	8.4	251	8.7	202	8.0	
Secondary school level	1,237	22.9	533	18.6	704	27.8	
University level	2,982	55.2	1,693	58.9	1,290	51.0	
Education level of father							<0.001
Never attend school	219	4.0	131	4.6	88	3.5	
Elementary school level	340	6.3	178	6.2	162	6.4	
Intermediate school level	519	9.6	274	9.6	245	9.7	
Secondary school level	1,287	23.8	617	21.5	670	26.5	
University level	3,036	56.2	1,672	58.2	1,364	54.0	
School type (*n* = 3,699; adolescents currently attending schools)							<0.001
Public school	2,294	62.0	1,261	56.6	1,033	70.2	
Private school	1,405	38.0	966	43.4	439	29.8	
Inclusion of nutrition education in schools’ curriculum (*n* = 3,699; adolescents currently attending schools)							<0.001
Yes	903	24.4	484	21.8	419	28.4	
No	2,796	75.6	1742	78.2	1,054	71.6	

**Figure 1:**
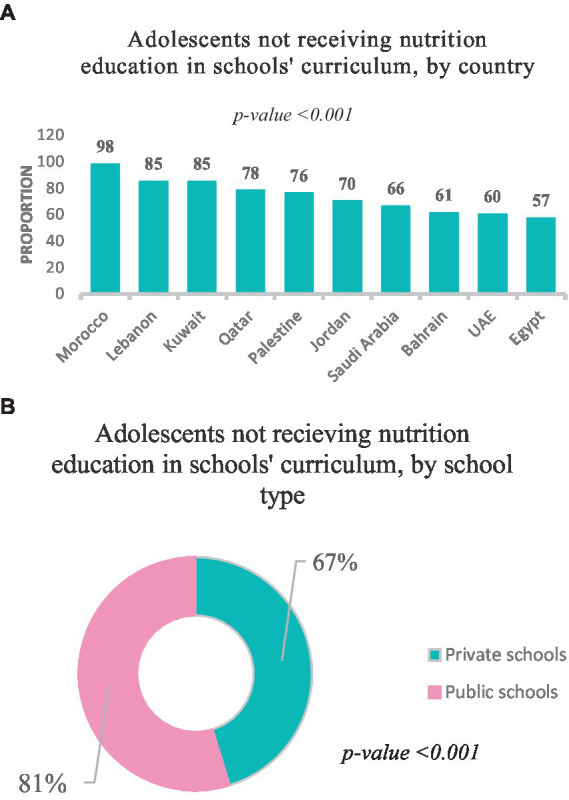
**(A)** Adolescents not receiving nutrition education in schools’ curriculum, by country (*n* = 3,699 school attendees). **(B)** Adolescents not receiving nutrition education in schools’ curriculum, by school type (*n* = 3,699 school attendees).

Among adolescents, 53.2% were males. The mean age ± SD of the adolescents was 15.9 ± 3.0. Moreover, 51% were in the late adolescence stage, whereas the remaining were either in the early (25%) or the middle (24%) adolescence stage, *p* < 0.001. Around 65% were of normal weight, 27% were overweight, 5% were underweight, and 3% were obese. In addition, 68.5 and 31% were school and university students, respectively. Also, 86% of the adolescents reported both parents as primary caregivers. Around 11.5% were currently working. About 76% were not receiving nutrition education as a part of their schools’ curriculum (Table 1). The highest proportion of adolescents who reported not receiving nutrition education was from Morocco (98%), followed by Lebanon (85%) andKuwait (85%), (Figure 1). Furthermore, 81% of adolescents attending public schools reported not receiving nutrition education, in contrast to 67% of those who were private school students, *p* < 0.001 (Figure 1).

As for the parent participants, 67.8% were mothers with a mean age ± SD of 43.0 ± 8.0. More than third of parents were overweight (fathers: 38.5%; mothers: 38.2%, p < 0.001). where39% of parents had no job, and half of themhad health problems (Table 1).

### 3.2. The level of adolescents’ nutrition literacy and the food literacy of their parents, overall and by country

In the overall adolescent population, the mean TNL score was 70.6 ± 9.5, with poor nutrition literacy was found in 28% of adolescents. The FNL, INL, and CNL scores were as follows: 22.7 ± 7, 18.5 ± 5.8, and 29.5 ± 4.6, respectively. Hence, 4 out of 10 adolescents (36%) were with poor FNL and INL, and 21% were with poor CNL (Table 2).

**Table 2:** General characteristics of parents, overall and by gender.

Parent participant	Overall (*n* = 5,401)		Father (*n* = 1739; 32.2%)		Mother (*n* = 3,661; 67.8%)		Value of *p*
	Mean ± SD		Mean ± SD		Mean ± SD		
Age: (Year)	45 ± 9.1		49 ± 10		43 ± 8		<0.001
	*n*	%	*n*	%	*n*	%	
Country of nationality							<0.001
Lebanon	594	11.0	201	11.6	392	10.7	
Bahrain	507	9.4	181	10.4	326	8.9	
Egypt	657	12.2	125	7.2	532	14.5	
Jordan	563	10.4	187	10.8	376	10.3	
Kuwait	483	8.9	98	5.6	385	10.5	
Morocco	590	10.9	379	21.8	210	5.7	
Palestine	517	9.6	142	8.2	375	10.2	
Qatar	549	10.2	135	7.8	414	11.3	
Saudi Arabia	491	9.1	136	7.8	355	9.7	
United Arab Emirates	450	8.3	154	8.9	296	8.1	
Weight status							<0.001
Underweight	121	2.2	47	2.7	74	2.0	
Normal-weight	1789	33.1	668	38.4	1,121	30.6	
Overweight	2069	38.3	670	38.5	1,399	38.2	
Obese	1,422	26.3	355	20.4	1,067	29.2	
Marital status							0.002
Married	4,885	90.5	1,605	92.3	3,280	89.6	
Divorced	284	5.2	65	3.8	218	218	
Widowed	232	4.3	69	4.0	163	163	
Number of children							<0.001
1	508	9.4	178	10.3	329	9.0	
2–3	2,790	51.7	974	56.0	1816	49.6	
>3	2,103	38.9	587	33.7	1,516	41.4	
Education level							<0.001
Never attend school	234	4.3	96	5.5	138	3.8	
Elementary school level	302	5.6	144	8.3	158	4.3	
Intermediate school level	355	6.6	113	6.5	242	6.6	
Secondary school level	1,198	22.2	410	23.6	788	21.5	
University level	3,312	61.3	976	56.1	2,336	63.8	
Spouses’ education level							<0.001
Never attend school	325	6.0	218	12.5	107	2.9	
Elementary school level	347	6.4	172	9.9	175	4.8	
Intermediate school level	383	7.1	111	6.4	272	7.4	
Secondary school level	1,244	23.0	407	23.4	836	22.8	
University level	3,102	57.4	831	47.8	2,271	62.0	
Job status							<0.001
No job	2,117	39.2	295	16.9	1822	49.7	
Full-time job	1851	34.3	820	47.2	1,031	28.2	
Part-time job	532	9.9	195	11.2	337	9.2	
Self-employed	901	16.7	429	24.7	472	12.9	
Prevalence of chronic diseases (*N* = 2,776 diseased parents; *n* males = 942; *n* females = 1834)							
Cardiovascular diseases (CVD)	230	8.3	90	9.5	140	7.6	0.02
Diabetes	603	21.7	264	28.0	339	18.5	<0.001
Hypertension	693	25.0	303	32.2	390	21.3	<0.001
Chronic kidney diseases (CKD)	91	3.3	36	3.8	55	3.0	0.13
Liver diseases	69	2.4	31	3.3	38	2.1	0.03
Osteoporosis	125	4.5	22	2.3	103	5.6	<0.001
Cancer	39	1.4	10	1.1	29	1.6	0.26
Respiratory diseases	199	7.2	65	7.0	134	7.3	0.89
Anemia	494	17.8	79	8.4	415	22.6	<0.001
Others	233	8.4	42	4.4	191	10.4	<0.001
Household crowding index*							
≤1^a^	3,196	59.2	–	–	–	–	–
>1^b^	1,011	18.7	–	–	–	–	–
>1.5^c^	1,194	22.1	–	–	–	–	–

*Number of co-residents (excluding newborns) divided by number of rooms (excluding kitchens and bathrooms). ^a^No crowding. ^b^Crowded. ^c^Over-crowded.

**Figure 2:**
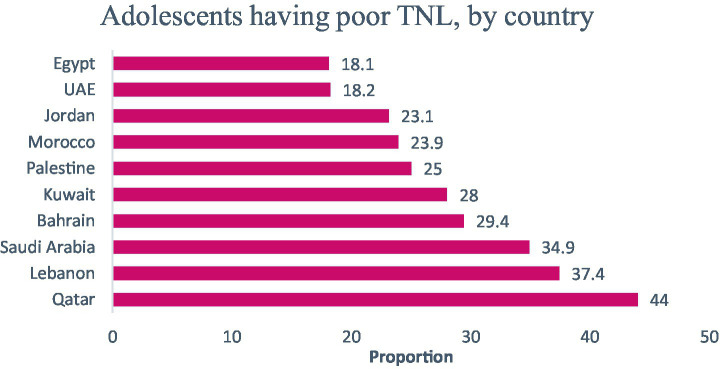
The proportion of adolescents having poor total nutrition literacy (TNL), by country.

As per country, Qatar and Lebanon showed the highest proportions of adolescents with poor TNL (44 and 37.4%, respectively), followed by Saudi Arabia (35%), Bahrain (29.4%), Kuwait (28%), Palestine (25%), Morocco (24%), Jordan (23%), UAE (18.2%), and Egypt (18.1%), *p* < 0.001 (Figure 2). Half the Egyptian adolescents (49.6%) were with poor FNL, followed by Qatari (41.7%) and Lebanese (39.4%) adolescents, *p* < 0.001 (Table 3). Adolescents participants with poor INL were mostly from Qatar (51%), followed by Lebanon (46%) and Bahrain (44.2%), *p* < 0.001 (Table 3). Similarly, around 3 out of 10 Saudi adolescents (29.4%) showed poor CNL, followed by Bahrain (24.2%) and Qatar (23.8%) *p* < 0.001 (Table 3). Regarding parents’ food literacy, an overall mean score of 32.8 ± 8.5 was reported, with 60% of parents were found with poor food literacy. Parents from Morocco (77%), Lebanon (71%), and Saudi Arabia (64%) were mostly food illiterate, followed by Qatar (59%), Jordan (58.7%), Palestine (55.3%), Kuwait (54.6%), UAE (52%), Bahrain (45.2%), and Egypt (42.7%), *p* < 0.001 (Table 3).

**Table 3:** The level of adolescents’ nutrition literacy and the food literacy of their parents, overall and by country.

	Overall (*N* = 5,401)	Lebanon (*n* = 594)	Bahrain (*n* = 507)	Egypt (*n* = 657)	Jordan (*n* = 563)	Kuwait (*n* = 483)	Morocco (*n* = 590)	Palestine (*n* = 517)	Qatar (*n* = 549)	Saudi Arabia (*n* = 491)	UAE (*n* = 450)	Value of *p*
	*n* (%)	*n* (%)	*n* (%)	*n* (%)	*n* (%)	*n* (%)	*n* (%)	*n* (%)	*n* (%)	*n* (%)	*n* (%)	
Adolescent: TNL^a^												
TNL score (Mean ± SD)	70.6 ± 9.5	69 ± 11	71 ± 11	68 ± 5	71 ± 8	72 ± 10	72 ± 10	72 ± 9	68 ± 9	70 ± 9	74 ± 10	<0.001*
Poor	1,514 (28.0)	222 (37.4)	150 (29.4)	119 (18.1)	130 (23.1)	135 (28.0)	141 (23.9)	130 (25.1)	242 (44.0)	171 (34.9)	82 (18.2)	<0.001**
Adequate	3,887 (72.0)	372 (62.6)	357 (70.6)	538 (81.9)	433 (76.9)	348 (72.0)	449 (76.1)	387 (74.9)	307 (56.0)	320 (65.1)	368 (81.8)	
Adolescent: FNL^b^												
FNL score (Mean ± SD)	22.7 ± 7	22.2 ± 6.7	24.9 ± 6.7	19 ± 7.7	23.1 ± 5.7	6.18 ± 22.3	22.4 ± 5.4	23.5 ± 6.7	22.3 ± 7.4	23.8 ± 7.1	25.3 ± 6.75	<0.001*
Poor	1919 (36)	234 (39.4)	139 (27.4)	326 (49.6)	173 (30.8)	191 (39.4)	209 (35.5)	154 (29.8)	229 (41.7)	159 (32.5)	110 (24.4)	<0.001**
Adequate	3,482 (64)	360 (60.6)	368 (72.6)	331 (50.4)	390 (69.2)	292 (60.6)	381 (64.5)	363 (70.2)	320 (58.3)	332 (67.5)	340 (75.6)	
Adolescent: INL^c^												
INL score (Mean ± SD)	18.45 ± 5.8	18 ± 6	18 ± 6	20 ± 6	19 ± 5	19 ± 6	19 ± 4	19 ± 6	17 ± 6	17 ± 6	19 ± 6	<0.001*
Poor	1944 (36.0)	273 (46.0)	224 (44.2)	88 (13.3)	165 (29.4)	166 (34.4)	200 (33.8)	185 (35.9)	279 (51.1)	210 (42.8)	153 (33.9)	<0.001**
Adequate	3,457 (64.0)	321 (54.0)	283 (55.8)	569 (86.7)	398 (70.6)	317 (65.6)	390 (66.2)	332 (64.1)	268 (48.9)	281 (57.2)	297 (66.1)	
Adolescent: CNL^d^												
CNL score (Mean ± SD)	29.46 ± 4.62	29 ± 5	29 ± 4	29 ± 4	29 ± 4	30 ± 4	31 ± 6	29 ± 4	29 ± 4	29 ± 5	30 ± 4	<0.001**
Poor	1,112 (20.6)	135 (22.6)	123 (24.2)	77 (11.7)	132 (23.5)	79 (16.3)	110 (18.7)	117 (22.8)	131 (23.8)	144 (29.4)	66 (14.6)	
Adequate	4,289 (79.4)	459 (77.4)	384 (75.8)	580 (88.3)	431 (76.5)	404 (83.7)	480 (81.3)	400 (77.2)	418 (76.2)	347 (70.6)	384 (85.4)	
Parent: FL^e^												
FL score (Mean ± SD)	32.8 ± 8.5	31 ± 9	35 ± 8	35 ± 9	32 ± 8	34 ± 7	29 ± 8	32 ± 9	33 ± 9	32 ± 8	35 ± 6	<0.001*
Poor	2,208 (59.7)	330 (70.8)	105 (45.2)	209 (42.7)	201 (58.7)	172 (54.6)	452 (77.0)	150 (55.3)	242 (59.0)	226 (63.8)	120 (52.2)	<0.001**
Adequate	1,492 (40.3)	138 (29.2)	128 (54.8)	281 (57.3)	142 (41.3)	143 (45.4)	135 (23.0)	121 (44.7)	168 (41.0)	128 (36.2)	110 (47.8)	

^a^Total Nutrition Literacy. ^b^Functional Nutrition Literacy. ^c^Interactive Nutrition Literacy. ^d^Critical Nutrition Literacy. ^e^Food Literacy.

* Significant at value of *p* < 0.05 of Kruskal-Wallis test.

** Significant at value of *p* < 0.05 of *χ*2 test.

### 3.3. The correlates of adolescents’ nutrition literacy in the overall sample population

Supplementary Table S1 shows the determinants of adolescents’ nutrition literacy. Most older adolescents (77.6%) had adequate TNL, compared to 63.6 and 68.2% of those in the young and middle stages respectively, *p* < 0.001. Similarly, FNL, INL, and CNL, were adequate among older adolescents compared to young and middle stage adolescents (72.5, 66.5, and 80%, respectively, all value of *p*s <0.001). In terms of adolescents’ gender, poor nutrition literacy was found significantly more among males compared to females; TNL (32.2% vs. 23.5%, *p* < 0.001), FNL (40.3% vs., 30.3%, *p* < 0.001), and INL (37.8% vs. 34%, *p* = 0.003), respectively. Poor nutrition literacy was also found more among obese adolescents; TNL (35.4%), FNL (43%), and INL (41.3%), although these findings were not significant (*p* = 0.17, *p* = 0.09, and *p* = 0.06, respectively). Parents who were underweight and with obesity had more adolescents with poor TNL (38 and 31.6%, respectively), compared to normal-weight and overweight parents (26.7 and 26.5%, respectively) *p* < 0.001. Further, it was noted that nearly half the obese parents (41.2%) had adolescent with poor FNL, *p* < 0.001. On the other hand, adequate level of nutrition literacy scores was found more among adolescents studying at university, with TNL (81%, *p* < 0.001), FNL (75%, p < 0.001), INL (68.7%, *p* < 0.001), and CNL (81%, *p* = 0.002), compared to those at school level (67.8, 59.5, 62, and 78.8%, respectively), and those not attending a school or university (50, 66.7%. 47.4, and 52.6%, respectively) (Supplementary Table S1).

Mothers with university degree had predominately adolescent with adequate INL (67%, *p* < 0.001) and CNL (81.6%, *p* < 0.001). Similarly, adolescents of fathers having university degree showed adequate TNL (72.2%, *p* = 0.012), FNL (63.5%, *p* = 0.008), INL (67%, *p* < 0.001), and CNL (80.4%, *p* < 0.001). In addition, adolescents having both parents as primary caregivers expressed adequate TNL (68.6%, *p* < 0.001), INL (58.8%, *p* = 0.03), and CNL (61.3%, *p* < 0.001). Moreover, working adolescents had predominately adequate TNL (79.8% vs. 70.8%, *p* < 0.001) and FNL (68.4% vs. 61.6%, *p* = 0.03), compared to non-workers. However, the school type was not a significant correlate except in the FNL, with adolescents in private schools scoring better in FNL compared to those in public schools (66% vs. 64.3%, *p* < 0.001). Adolescents receiving nutrition education showed significantly better TNL and INL in contrast to others who did not report so (73.6% vs. 65.9%, *p* < 0.001) and (68.5% vs. 59.8%, *p* < 0.001), respectively. Most married parents had adolescent with adequate TNL (68.6%, *p* < 0.001), INL (61.5%, *p* < 0.001), and CNL (79.4%, p < 0.001). Moreover, parents having 2–3 children had a higher proportion of adolescent with adequate TNL (69.4%, *p* = 0.015), INL (65%, *p* < 0.001), and CNL (82%, *p* < 0.001). Around 36% of parents with reported disease (vs. 18% healthy) had adolescent with poor TNL, *p* = 0.017. As well, a higher proportion of food illiterate parents (vs. food literate) had adolescent who were nutritionally illiterate too, in TNL (36.6% vs. 25.8%, *p* < 0.001) INL (45.2% vs. 27.5%, *p* < 0.001) and CNL (25.3% vs. 15%, *p* < 0.001) dimensions (Supplementary Table S1).

### 3.4. Predictors of the adolescents’ nutrition literacy in this study

Table 4 shows the significant predictors of adolescents’ nutrition literacy. Older adolescents (vs. younger adolescents) were 1.6 times (OR = 1.6, CI = 1.4–1.9, *p* < 0.001) more likely to be nutritionally literate. Female adolescents had a 30% more probability of having adequate nutrition literacy (vs. males OR = 0.7, CI = 0.6–0.8, *p* < 0.001). Besides, adolescents who were university students (OR = 4.5, CI = 1.8–11.5, *p* = 0.001) were 4.5 times more likely to be nutritionally literate. Adolescents with parents who were either overweight or obese were 1.5 times more likely to be with adequate nutrition literacy (OR = 1.5, CI = 1.0–2.2, *p* = 0.04). Further, adolescents with both parents as primary caregivers (vs. either parent and others, OR = 0.7, C I = 0.6–0.8, *p* < 0.001, and OR = 0.7, CI = 0.5–1, *p* = 0.03, respectively) were 30, and 60% (vs. living alone, OR = 0.4, CI = 0.1, 1.1, *p* = 0.001) more likely to be nutritionally literate. Furthermore, working adolescents were 1.5 times more likely to have adequate nutrition literacy (OR = 1.5, CI = 1.2–1.8, 0.001). Parents with one child (vs. ≥ 3 children) were 30% more likely to be with adequate nutrition literacy (OR = 0.7, CI = 0.5–0.9, *p* < 0.001). Adolescents receiving nutrition education were 30% more likely to be nutritionally literate (OR = 1.3, CI = 1.1–1.5, *p* = 0.01). Parents who reported to be healthy were 20% more likely to have adolescent who are nutritionally literate (OR = 1.2, CI = 1.0–1.4, *p* = 0.01). In addition, food literate parents were 2 times more likely to have nutritionally literate adolescent (OR = 1.8, CI = 1.6–2.1, *p* < 0.001) (Table 4).

**Table 4:** Predictors factors of adolescents’ nutrition literacy.

Binary logistic regression taking the total nutrition literacy (TNL) [poor (reference) vs. adequate] as the dependent variable	AOR (95% CI)	Value of *p*
Adolescence stage reference: Early (10–13 years old)	–	–
Middle (14–16 years old)	1.3 (1.1–1.5)	0.003
Late (17–19 years old)	1.6 (1.4–1.9)	<0.001
Gender (adolescent) reference: Females	–	–
Males	0.7 (0.6–0.8)	<0.001
Adolescent education level (Reference: Not attending school or university)		
University level	4.5 (1.8–11.5)	0.001
Parental weight status reference: Underweight	–	–
Overweight/Obese	1.5 (1.0–2.2)	0.04
Primary caregiver (Reference: Both parents)		
Either parent	0.7 (0.6–0.8)	<0.001
Others	0.7 (0.5–1.0)	0.03
None (living alone)	0.4 (0.1–1.1)	0.001
The adolescent is currently working (Reference: No)		
Yes	1.5 (1.2–1.8)	0.001
Number of children in the household (Reference: One child)		
2–3 children	0.9 (0.7–1.1)	0.37
More than 3	0.7 (0.5–0.9)	0.001
The adolescent is receiving nutrition education in their schools’ curriculum (Reference: No)		
Yes	1.3 (1.1–1.5)	0.01
Parent has one or more chronic disease (Reference: Yes)		
No	1.2 (1.0–1.4)	0.01
Parental food literacy (Reference: Poor)		
Adequate	1.8 (1.6–2.1)	<0.001

## 4. Discussion

This study assessed the Arab adolescents’ nutrition literacy and the food literacy of their parents. About 28% of adolescents had poor nutrition literacy, with 60% of their parents being food “less literate.” Nutrition illiteracy was most prevalent in Qatar (44%), Lebanon (37.4%), and Saudi Arabia (34.9%). Adolescents’ age, gender, education level, primary caregiving, employment status, and receiving nutrition education in schools predicted their nutrition literacy levels. Besides, parental weight status, health status, parent food literacy level, and the number of children per household were significant determinants.

There is scarcity of studies evaluating nutrition literacy among the Arab population. All in all, only three studies on this topic were conducted and were chiefly in Lebanon and Palestine (16, 23, 24). Our study findings are in concordance with that previously reported in Lebanon which highlight inadequate nutrition literacy status among adolescents (23), but in high contrast to that observed among Palestinian adults (75% were nutritionally illiterate) (24). Apart from the Arab region, our observed mean score ± SD of nutrition literacy (70.6 ± 9.5) is quite close to that reported among a study in China (61.7 ± 14.37) (25) and 3 studies in Turkey (67.6 ± 7.9; 72.3 ± 8.2; 70.31 ± 8.6) (17, 26, 27). In the present study, Qatari adolescents pose an added risk of nutrition illiteracy (44%). A review of studies in Qatar shows that the high gross domestic product has led to the adoption of western lifestyles in the country, promoting childhood overweight and obesity (28). Besides, the highest levels of obesity among Arab countries were observed in Bahrain, Kuwait, Qatar, and UAE (29). Nutrition literacy is a critical influencing factor of obesity, with a higher literacy level associated with appropriate dietary habits and food-related behaviors (30). Lebanon, on the other hand, ranked second in the present study for adolescent nutrition illiteracy. Between 1997 and 2009, significant changes were observed in the diet of the Lebanese population, with high intakes of salty snacks, added fats, and oils (31). This dietary transition, along with the increase in sedentary behaviors, poor nutrition knowledge, and the economic collapse, has put Lebanon at risk of further constraints in terms of malnutrition prevalence (31). In this context, our study shows that 75.6% of adolescents do not receive nutrition education in schools. Further, nutrition education was mostly provided for Egyptian adolescents who were mostly nutritionally literate. Hence, the latter finding reemphasize the relevance of nutrition education in improving students’ nutrition literacy. In this regard, several school-based nutrition education programs have shown effectiveness in promoting nutrition knowledge, healthy dietary habits, and self-efficacy among students (32–35). Therefore, such interventions are indispensable in the Arab region where malnutrition prevalence is of mounting concern. In addition to classroom approaches, farm-to-school programs, school gardens, and cooking programs might be also prioritized (19). In this study, adolescents of older age reported better nutrition literacy levels than younger ones. Variations in nutrition literacy levels among adolescence age could be due to increased exposure to and interest in health-related information with advancing age (36). Furtherly, female adolescents were more nutritionally literate than males. This is consistent with data from Turkey (17, 27) and Iran (15, 37). One possible explanation for gender disparities is that females focus on the nutritional value of food and prioritize healthy eating more than men (38). Women have better nutrition knowledge, and recognize nutrition as a critical contributor to their conception of health (39). Furthermore, higher education levels determined better adolescents’ nutrition literacy in our study, which is consistent with data from China (25) and Italy (40). Individuals with the highest education level have a better ability to understand, process, and apply nutrition information. Our findings also showed that working adolescents had better nutrition literacy than their counterparts. We believe that adolescent workers are most likely employed in surroundings that promote health and nutrition. In addition, adolescents having both parents as primary caregivers had the most adequate nutrition literacy, which goes hand in hand with findings from China (25). Above all, obese and overweight parents had more nutritionally illiterate adolescent than their counterparts. Obesity among parents might drive their children to attain more nutrition information to help them change their dietary habits and lose or maintain body weight. Diseased parents in the current study had mostly nutritionally illiterate adolescent. These findings suggest that the occurrence of nutrition-related diseases among parents, which usually correlates with unhealthy diets and lack nutrition knowledge, may negatively affect their children’s nutrition literacy. Moreover, food literate parents had mostly nutritionally literate adolescent. This finding is supported by those reported in Greece (41) and the United States (42), where parental nutrition literacy was positively correlated with healthy parental feeding practices (PFP), and child Healthy Eating Index (HEI). Food literate parents are more likely to engage their children in nutrition communication and expose them to reliable information sources about nutrition from professionals (43). Our data may be useful reference for policymakers and curriculum developers to assess education and develop practical learning and teaching strategies to improve student’s food and nutrition literacy as indicated in a recent systematic review (44).

### 4.1. Limits and strengths

The current study had some limitations that should be acknowledged. First, due to the unavailability of valid questionnaires regarding nutrition and food literacy in the region, the questionnaires in this study were derived from many credible, valid sources and translated to Arabic then back translated to English by experts. Second, due to its cross-sectional design, causal inferences cannot be drawn. Third, the self-administered questionnaire causes inevitable information bias. Fourth, convenience sampling could lead to skewed sample characteristics. Fifth, we collected no information on adolescents’ food habits which most probably correlate with nutrition literacy. Nonetheless, this study is the first of the region’s kind, with a large sample of Arab adolescents and their parents addressing the nutrition and food literacy topics.

## 5. Conclusion

This study shows that nutrition literacy inadequacy among Arab adolescents is a prioritized challenge to be addressed. The “macro-curriculum” concept in schools includes intervention packages to implement, leading to the best possible nutrition outcomes. The target group for interventions includes school-age children, teachers at both public and private institutions, as well as the ministries of education and health in each Arab nation. Classroom activities, such as counting with pictures of fruits and vegetables, learning about cultural food traditions, and measuring ingredients for a recipe, could be included in school interventions. Additionally, schools can send daily messages with nutrition content, such as morning announcements, to other family members. Staff meetings, parent-teacher interviews, and home cooking activities are also things to be considered.

## Data availability statement

The raw data supporting the conclusions of this article will be made available by the authors, without undue reservation.

## Ethics statement

The study was conducted in accordance with the Declaration of Helsinki, and approved by the Ethics Committee of Al Zahraa University Medical Center (ZhU#17, 2022), Beirut, Lebanon, and the universities from all participating countries. Written informed consent to participate in this study was provided by the participants’ legal guardian/next of kin.

## Regional food literacy group

Bahrain: Tariq Abdulkarim Alalwan; Palestine: Malak Amro; Qatar: Aljazi AlQahtani; United Arab Emirates: Leila Cheikh Ismail.

## Author contributions

MH: conceptualization, validation, and project administration. HM, KB, FH, SA, DA, HA, HB, IK, RQ, and RT: methodology. RQ: software. HM: formal analysis. MH and HM: data curation and writing—original draft preparation. All authors: writing—review and editing. All authors have read and agreed to the published version of the manuscript.

## Funding

Open access funding provided by the Qatar National Library.

## Conflict of interest

The authors declare that the research was conducted in the absence of any commercial or financial relationships that could be construed as a potential conflict of interest.

## Publisher’s note

All claims expressed in this article are solely those of the authors and do not necessarily represent those of their affiliated organizations, or those of the publisher, the editors and the reviewers. Any product that may be evaluated in this article, or claim that may be made by its manufacturer, is not guaranteed or endorsed by the publisher.

## Footnotes

^1^ https://survey.emfid.org/
