# Supplementary material for: Status and correlates of food and nutrition literacy among parents-adolescents’ dyads: findings from 10 Arab countries
Source: Front Nutr. 2023 May 2;10:1151498. doi: 10.3389/fnut.2023.1151498 (PMC10186151; doi:10.3389/fnut.2023.1151498)
Supplement: Supplementary file 1 [file Table_1.DOCX]

**Table S1.** The correlates of adolescents’ nutrition literacy in the overall sample population.

|  | TNL ^(a)^ | | p-value ^(e)^ | FNL ^(b)^ | | p-value  ^(f)^ | INL ^(c)^ | | p-value  ^(g)^ | CNL ^(d)^ | | p-value ^(h)^ |
| --- | --- | --- | --- | --- | --- | --- | --- | --- | --- | --- | --- | --- |
|  | Poor | Adequate |  | Poor | Adequate |  | Poor | Adequate |  | Poor | Adequate |  |
|  | n (%) | n (%) |  | n (%) | n (%) |  | n (%) | n (%) |  | n (%) | n (%) |  |
| **Adolescence stage** |  |  | <0.001* |  |  | <0.001* |  |  |  |  |  | <0.001* |
| Early adolescence (10-13 years old) | 493 (36.4) | 861  (63.6) |  | 654  (48.3) | 699  (51.7) |  | 548  (40.5) | 805  (59.5) | <0.001* | 246  (18.1) | 1107  (81.9) |  |
| Middle adolescence (14-16 years old) | 413 (31.8) | 886  (68.2) |  | 513  (39.5) | 786  (60.5) |  | 477  (36.7) | 823  (63.3) |  | 316  (20.0) | 984  (80.0) |  |
| Late adolescence (17-19 years old) | 616 (22.4) | 2132  (77.6) |  | 757  (27.5) | 1991  (72.5) |  | 920  (33.5) | 1828  (66.5) |  | 552  (20.0) | 2196  (80.0) |  |
| **Adolescent’s gender** |  |  | <0.001* |  |  | <0.001* |  |  | 0.003* |  |  | 0.154 |
| Female | 593 (23.5) | 1935  (76.5) |  | 766  (30.3) | 1763  (69.7) |  | 859  (34.0) | 1670  (66.0) |  | 500  (19.7) | 2029  (80.3) |  |
| Male | 928 (32.3) | 1944  (67.7) |  | 1158 (40.3) | 1714  (59.7) |  | 1086  (37.8) | 1786  (62.2) |  | 613  (21.3) | 2259  (78.7) |  |
| **Weight status of adolescent** |  |  | 0.17 |  |  | 0.09 |  |  | 0.06 |  |  | 0.06 |
| Underweight | 73  (27.7) | 190  (72.3) |  | 94  (35.7) | 169  (64.3) |  | 77  (29.2) | 186  (70.8) |  | 43 (16.4) | 219  (83.6) |  |
| Normal weight | 965 (27.8) | 2506  (72.2) |  | 1245  (35.8) | 2227  (64.2) |  | 1258  (36.2) | 2214  (63.8) |  | 709  (20.4) | 2762  (79.6) |  |
| Overweight | 400  (27.6) | 1051  (72.4) |  | 490  (33.7) | 961  (66.3) |  | 519  (35.7) | 932  (64.3) |  | 322  (22.2) | 1129  (77.8) |  |
| Obese | 63  (35.4) | 115  (64.6) |  | 77  (43.0) | 102  (57.0) |  | 74  (41.3) | 105  (58.7) |  | 31  (17.3) | 148  (82.7) |  |
| **Parental weight status** |  |  | <0.001* |  |  | <0.001* |  |  | 0.047* |  |  | <0.001* |
| Underweight | 46  (38) | 75  (62) |  | 33  (27.2) | 88  (72.8) |  | 48  (39.6) | 73  (60.4) |  | 42  (34.7) | 79  (65.3) |  |
| Normal weight | 477  (26.7) | 1311  (73.3) |  | 579  (32.4) | 1210  (67.6) |  | 683  (38.2) | 1105  (61.8) |  | 414  (23.1) | 1374  (76.9) |  |
| Overweight | 548  (26.5) | 1521  (73.5) |  | 726  (35.1) | 1343  (64.9) |  | 733  (35.4) | 1336  (64.6) |  | 386  (18.6) | 1683  (81.4) |  |
| Obese | 450  (31.6) | 973  (68.4) |  | 586  (41.2) | 836  (58.8) |  | 480  (33.7) | 943  (66.3) |  | 271  (19.0) | 1152  (81.0) |  |
| **Education level of adolescents** |  |  | <0.001* |  |  | <0.001* |  |  | <0.001* |  |  | 0.002* |
| Not attending school | 9  (50.0) | 9  (50.0) |  | 6  (33.3) | 12  (66.7) |  | 10  (52.6) | 9  (47.4) |  | 9  (47.4) | 10  (52.6) |  |
| School level | 1194  (32.2) | 2508  (67.8) |  | 1499  (40.5) | 2203  (59.5) |  | 1408  (38.0) | 2294  (62.0) |  | 786  (21.2) | 2915  (78.8) |  |
| University level | 318  (18.9) | 1363  (81.1) |  | 419  (25.0) | 1261  (75.0) |  | 527  (31.3) | 1154  (68.7) |  | 318  (18.9) | 1363  (81.1  ) |  |
| **Education level of mother** |  |  | 0.08 |  |  | 0.476 |  |  | <0.001* |  |  | <0.001* |
| Never attend school | 111  (33.5) | 220  (66.5) |  | 124  (37.5) | 206  (62.5) |  | 141  (42.7) | 189  (57.3) |  | 85  (25.7) | 245  (74.3) |  |
| School level | 584  (28.0) | 1504  (72.0) |  | 725  (34.7) | 1362  (65.3) |  | 818  (39.1) | 1270  (60.9) |  | 479  (22.9) | 1609  (77.1) |  |
| University level | 826  (27.7) | 2156  (72.3) |  | 1075  (36.0) | 1908  (64.0) |  | 985  (33.0) | 1997  (67.0) |  | 549  (18.4) | 2433  (81.6) |  |
| **Education level of father** |  |  | 0.012* |  |  | 0.008* |  |  | <0.001* |  |  | <0.001* |
| Never attend school | 81  (36.9) | 138  (63.1) |  | 92  (42.2) | 126  (57.8) |  | 102 (46.5) | 117  (53.5) |  | 68  (31.2) | 150  (68.8) |  |
| School level | 594  (27.7) | 1552  (72.3) |  | 719  (33.5) | 1427  (66.5) |  | 839  (39.0) | 1307  (61.0) |  | 450  (21.0) | 1695  (79.0) |  |
| University level | 846  (27.8) | 2190  (72.2) |  | 1113  (36.6) | 1923  (63.4) |  | 1004  (33.0) | 2033  (67.0) |  | 595  (19.6) | 2442  (80.4) |  |
| **Primary caregiver** |  |  | 0.001* |  |  | 0.112 |  |  | 0.033* |  |  | <0.001* |
| Both parents | 1015  (31.4) | 2216  (68.6) |  | 1330  (41.2) | 1900  (58.8) |  | 1330  (41.2) | 1900  (58.8) |  | 1250  (38.7) | 1980  (61.3) |  |
| Either parent | 144  (36.2) | 254  (63.8) |  | 144  (36.2) | 254  (63.8) |  | 144  (36.2) | 254  (63.8) |  | 130  (32.6) | 268  (67.4) |  |
| Others | 33  (47.8) | 36  (52.2) |  | 24  (34.8) | 45  (65.2) |  | 24  (34.8) | 45  (65.2) |  | 26  (37.7) | 43  (62.3) |  |
| None (living alone) | 2  (100.0) | 0  (0) |  | 0  (0) | 2  (100.0) |  | 0  (0) | 2  (100.0) |  | 2  (100.0) | 0  (0) |  |
| **The adolescent is currently working** |  |  | <0.001* |  |  | 0.03* |  |  | 0.10 |  |  | 0.56 |
| No | 1396  (29.2) | 3386  (70.8) |  | 1728  (36.1) | 3054  (61.6) |  | 1740  (36.5) | 3024  (61.5) |  | 980  (20.5) | 3802  (79.5) |  |
| Yes | 125  (20.2) | 493  (79.8) |  | 196  (31.6) | 423  (68.4) |  | 204  (33.0) | 414  (77.0) |  | 133  (21.5) | 486  (78.5) |  |
| **School type** |  |  | 0.81 |  |  | 0.001* |  |  | 0.45 |  |  | 0.67 |
| Public | 722  (31.5) | 1572  (68.4) |  | 880  (38.4) | 1414  (64.3) |  | 884  (38.5) | 1410  (61.5) |  | 492  (21.4) | 1802  (78.6) |  |
| Private | 472  (33.6) | 933  (66.4) |  | 619  (44.0) | 787  (66.0) |  | 524  (37.3) | 881  (62.7) |  | 293  (21.0) | 1112  (79.0) |  |
| **Inclusion of nutrition education in schools’ curriculum** |  |  | <0.001* |  |  | 0.22 |  |  | <0.001* |  |  | 0.06 |
| No | 955  (34.1) | 1841  (65.9) |  | 1117  (40.0) | 1679  (60.0) |  | 1123  (40.2) | 1672  (59.8) |  | 613  (21.9) | 2183  (78.1) |  |
| Yes | 239  (26.4) | 665  (73.6) |  | 382  (42.3) | 522  (57.7) |  | 284  (31.5) | 619  (68.5) |  | 172  (19.0) | 731  (81.0) |  |
| **Parental marital status** |  |  | <0.001* |  |  | 0.094 |  |  | <0.001* |  |  | <0.001* |
| Married | 1052  (31.4) | 2299  (68.6) |  | 1360  (40.8) | 1992  (59.2) |  | 1291  (38.5) | 2060  (61.5) |  | 691  (20.6) | 2660  (79.4) |  |
| Divorced | 77  (37) | 131  (63) |  | 92  (44.4) | 115  (55.6) |  | 54  (26.0) | 153  (74.0) |  | 47  (22.6) | 161  (77.4) |  |
| Widowed | 64  (45.7) | 76  (54.3) |  | 46  (32.8) | 94  (67.2) |  | 62  (44.2) | 78  (55.8) |  | 47  (33.3) | 94  (66.7) |  |
| **Number of children at the household** |  |  | 0.015* |  |  | 0.144 |  |  | <0.001* |  |  | <0.001* |
| 1 | 104  (31.3) | 228  (68.7) |  | 121  (36.3) | 212  (63.7) |  | 125  (37.5) | 208  (62.5) |  | 93  (28.0) | 240  (72.0) |  |
| 2-3 | 649  (30.6) | 1472  (69.4) |  | 883  (41.6) | 1238  (58.4) |  | 745  (35.1) | 1376  (64.9) |  | 380  (18.0) | 1741  (82.0) |  |
| More than 3 | 441  (35.4) | 805 (64.6) |  | 494  (39.7) | 751  (60.3) |  | 538  (43.2) | 708  (56.8) |  | 311  (25.0) | 934  (75.0) |  |
| **Parent has one or more chronic disease** |  |  | 0.017* |  |  | 0.832 |  |  | 0.561 |  |  | **0.91** |
| No | 473  (18) | 2150  (82) |  | 787 (30) | 1835  (70) |  | 1010  (38.5) | 1613  (61.5) |  | 1042  (39.7) | 1581  (60.3) |  |
| Yes | 1040 (37.5) | 1737  (62.5) |  | 1131  (40.7) | 1644  (59.3) |  | 1133  (40.8) | 1644  (59.2) |  | 872  (31.4) | 1905  (68.6) |  |
| **Parental food literacy** |  |  | <0.001* |  |  | 0.208 |  |  | <0.001* |  |  | <0.001* |
| Poor | 808 (36.6) | 1400 (63.4) |  | 913  (41.3) | 1295  (58.7) |  | 997  (45.2) | 1210  (54.8) |  | 559  (25.3) | 1649  (74.7) |  |
| Adequate | 386 (25.8) | 1105  (74.2) |  | 586  (39.3) | 906  (60.4) |  | 411  (27.5) | 1081  (72.5) |  | 226  (15.0) | 1266  (85.0) |  |

1. Total Nutrition Literacy
2. Functional Nutrition Literacy
3. Interactive Nutrition Literacy
4. Critical Nutrition Literacy
5. Significance level related to TNL
6. Significance level related to FNL
7. Significance level related to INL
8. Significance level related to CNL

* significant p-value < 0.05 for χ 2 test
